# Fabrication and Characterization of Porous PEGDA Hydrogels for Articular Cartilage Regeneration

**DOI:** 10.3390/gels10070422

**Published:** 2024-06-26

**Authors:** Silvia Gonella, Margarida F. Domingues, Filipe Miguel, Carla S. Moura, Carlos A. V. Rodrigues, Frederico Castelo Ferreira, João C. Silva

**Affiliations:** 1 Department of Bioengineering, iBB—Institute for Bioengineering and Biosciences, Instituto Superior Técnico, Universidade de Lisboa, Av. Rovisco Pais, 1049-001 Lisboa, Portugal; gonel.silvia@gmail.com (S.G.); margarida.domingues@tecnico.ulisboa.pt (M.F.D.); filipearmiguel1999@gmail.com (F.M.); carlos.rodrigues@tecnico.ulisboa.pt (C.A.V.R.); frederico.ferreira@tecnico.ulisboa.pt (F.C.F.); 2 Associate Laboratory i4HB—Institute for Health and Bioeconomy, Instituto Superior Técnico, Universidade de Lisboa, Av. Rovisco Pais, 1049-001 Lisboa, Portugal; 3 Polytechnic Institute of Coimbra, Applied Research Institute, Rua da Misericórdia, Lagar dos Cortiços—S. Martinho do Bispo, 3045-093 Coimbra, Portugal; carla.moura@ipc.pt; 4 CDRSP—Centre for Rapid and Sustainable Product Development, Polytechnic of Leiria, Rua de Portugal-Zona Industrial, 2430-028 Marinha Grande, Portugal; 5 Research Centre for Natural Resources Environment and Society (CERNAS), Polytechnic Institute of Coimbra, Bencanta, 3045-601 Coimbra, Portugal

**Keywords:** articular cartilage tissue engineering, biocompatibility tests, chondrogenic differentiation, gas foaming, hydrogels, mesenchymal stem/stromal cells, polyethylene glycol diacrylate

## Abstract

Functional articular cartilage regeneration remains an unmet medical challenge, increasing the interest for innovative biomaterial-based tissue engineering (TE) strategies. Hydrogels, 3D macromolecular networks with hydrophilic groups, present articular cartilage-like features such as high water content and load-bearing capacity. In this study, 3D porous polyethylene glycol diacrylate (PEGDA) hydrogels were fabricated combining the gas foaming technique and a UV-based crosslinking strategy. The 3D porous PEGDA hydrogels were characterized in terms of their physical, structural and mechanical properties. Our results showed that the size of the hydrogel pores can be modulated by varying the initiator concentration. In vitro cytotoxicity tests showed that 3D porous PEGDA hydrogels presented high biocompatibility both with human chondrocytes and osteoblast-like cells. Importantly, the 3D porous PEGDA hydrogels supported the viability and chondrogenic differentiation of human bone marrow-derived mesenchymal stem/stromal cell (hBM-MSC)-based spheroids as demonstrated by the positive staining of typical cartilage extracellular matrix (ECM) (glycosaminoglycans (GAGs)) and upregulation of chondrogenesis marker genes. Overall, the produced 3D porous PEGDA hydrogels presented cartilage-like mechanical properties and supported MSC spheroid chondrogenesis, highlighting their potential as suitable scaffolds for cartilage TE or disease modelling strategies.

## 1. Introduction

Articular cartilage (AC) is a specialized connective tissue that covers the bone extremities within diarthrodial joints [1]. Its primary functions include protecting the underlying subchondral bone from mechanical damage [2] and providing a lubricated surface conducive to smooth articulation and minimized friction [1,3]. AC comprises a sparse population of specialized cells, the chondrocytes, surrounded by a dense extracellular matrix (ECM), devoid of blood/lymphatic vessels and nerves [4,5]. Predominantly composed of water, the ECM facilitates nutrient transport to chondrocytes and acts as a lubricant [1], harboring structural macromolecules, such as collagen type II, proteoglycans, non-collagenous proteins and glycoproteins [4]. Chondrocytes, derived from mesenchymal stem/stromal cells (MSCs), play a crucial role in maintaining tissue homeostasis by synthesizing ECM components, growth factors and enzymes in response to various stimuli, albeit presenting low metabolic activity [1]. AC exhibits four distinct zones, each characterized by varying ECM composition and chondrocyte content. The superficial zone, in contact with the synovial fluid, contributes to the tensile properties of AC, presenting high chondrocyte density; conversely, the middle and deep zones primarily withstand compressive forces, containing elevated levels of proteoglycans, while the calcified zone secures the collagen fibrils to the subchondral bone [1].

In the event of an injury, the absence of blood vessels and the low metabolic rate of chondrocytes hinder the delivery of oxygen, nutrients and immune cells and the production of new cartilage, thus compromising the tissue’s self-healing ability [3,6,7]. Consequently, if left untreated, injuries to AC, whether acute or chronic traumatic events, are often unresolved and can progress to osteoarthritis (OA) [8]. OA, a chronic degenerative joint disease, primarily affects the elderly population and is characterized by AC degradation, synovial inflammation and subchondral bone thickening [3,6]. It mainly manifests in the knees, hip, spine and hands [3,9], featuring symptoms like activity-exacerbated pain, deformity and reduced range of motion [3]. Although the pathogenesis of OA remains elusive, evidence suggests an imbalance in tissue remodeling [4], possibly triggered by chondrocyte apoptosis induced by oxidative stress and mechanical signals [10]. In 2019, approximately 528 million individuals worldwide suffered from OA, of which 10 and 18% were men and women over 60 years of age, respectively [11,12]. With an ageing population and the rising prevalence of risk factors such as obesity, this number is expected to continue to increase in the future [13]. Current treatments for OA, including non-surgical interventions such as physical therapy and medication, as well as surgical procedures (e.g., microfracture, mosaicplasty, autologous chondrocyte implantation (ACI)) offer limited efficacy, which has prompted the development of alternative approaches [7]. Regenerative medicine, for instance, which comprises cell therapy and tissue engineering (TE), is currently being explored for AC defect repair and regeneration [3].

Cartilage TE aims to promote effective regeneration of damaged cartilage through the development and in vivo implantation of functional biomimetic scaffolds combined with cells, growth factors and other biochemical/physical cues [14]. These constructs are meticulously designed to replicate the structural/compositional properties of native AC and provide an optimal environment for chondrocyte growth and ECM production [6]. Cartilage TE requires careful consideration regarding the scaffold material (natural or synthetic), scaffold type (e.g., 3D-printed structures, sponges, nanofibrous matrices, hydrogels), cell types (e.g., chondrocytes, MSCs) and growth factors/small molecules (e.g., transforming growth factor-β (TGF-β), kartogenin) to include [14,15,16,17]. Besides their capacity to differentiate into chondrocytes, MSCs are a promising source for cartilage TE strategies due to their high availability for isolation from several human tissues (e.g., adipose tissue, bone marrow, synovial membrane, umbilical cord), high in vitro proliferation capacity, low immunogenicity and favorable immunomodulatory/trophic properties [18,19,20]. The scaffold material must exhibit biocompatibility, biodegradability (essential for in vivo implantation) and mechanical properties similar to the native AC tissue, supporting chondrogenesis [5]. 

Hydrogels are crosslinked 3D macromolecular networks that possess high water content, making them promising scaffold types for cartilage TE [14]. Hydrogels can be produced from natural biomaterials, such as collagen and hyaluronic acid—naturally present in AC—or from synthetic materials, such as polyglycolic acid (PGA), polylactic acid (PLA) and polyethylene glycol (PEG), which can be tailored to attain specific mechanical properties [5]. Among synthetic hydrogels, polyethylene glycol diacrylate (PEGDA), synthesized via PEG acrylation and photopolymerizable with UV light, is particularly attractive for cartilage regeneration: PEGDA hydrogels exhibit high hydrophilicity, resembling the aqueous AC environment, and can achieve a compressive modulus comparable to native cartilage (790 ± 360 kPa) [21,22]. Furthermore, PEGDA scaffolds have been shown to sustain TGF-β-mediated chondrogenesis of undifferentiated cells, such as MSCs [23]. Nonetheless, due to its hydrophilic nature and highly mobile backbone, PEGDA provides non-adhesive surfaces, resulting in limited protein adsorption and cell adhesion, which is undesirable for several TE applications [24]. The incorporation of cell adhesive ligands, including RGD motifs, into PEGDA matrices has been reported to address this issue, with results demonstrating the adhesion of different cell types to PEGDA-based hydrogels [25,26,27]. In addition to mechanical properties, hydrogels for cartilage TE should possess adequate porosity to facilitate nutrient diffusion, thereby supporting chondrocyte survival, proliferation and migration within the 3D structure [14]. Several techniques have been used to control hydrogel porosity, including solvent casting, freeze-drying, gas foaming and micropatterning [28]. Gas foaming, in particular, stands out as a cost-effective method for introducing porosity in hydrogels, capable of yielding porosities up to 90% [21,29]. It involves the generation of gaseous porogens within a polymer during polymerization, which become entrapped inside the crosslinked polymer, resulting in a porous structure conducive to cell infiltration [30]. Foaming agents are incorporated into the prepolymer solution, releasing gases either through chemical reactions or by the addition of inert gases at low/high pressures [31]. For instance, sodium bicarbonate, the most used foaming agent, releases CO_2_ in mildly acidic conditions [21]. 

In this study, we developed 3D porous PEGDA hydrogels using a combination of gas foaming and ultraviolet (UV) light-based polymerization, aiming to achieve similar mechanical properties to articular cartilage tissue and to support chondrogenesis. While similar methodologies have been employed to fabricate porous PEG-based hydrogels for TE strategies [32,33,34,35], none have been specifically designed for AC TE. The novelty of this study lies on the use of these 3D highly porous interconnected PEGDA hydrogels to culture MSC spheroids under chondrogenic differentiation conditions, envisaging cartilage TE or disease (e.g., OA) modelling applications.

## 2. Results and Discussion

### 2.1. Production and Structural Characterization of 3D Porous PEGDA Hydrogels

Three-dimensional porous PEGDA hydrogels were fabricated by combining gas foaming and UV-induced crosslinking. Gas foaming commonly requires mixing foaming agents with a prepolymer solution, leading to the release of gas bubbles that become trapped within the hydrogel matrix during polymerization, resulting in porous structures. While polymerization is often induced by non-photocurable initiators or temperature changes [32,36,37], in this study, UV-based crosslinking was the chosen method, with sodium bicarbonate and acetic acid serving as the foaming agents. Sodium bicarbonate powder was dispersed on the bottom of a well, followed by the addition of a 20% *v*/*v* PEGDA solution with the photoinitiator. Subsequently, acetic acid was added, and the plate was exposed to UV light, which allowed simultaneous gas foaming, triggered by the reaction between acetic acid and sodium bicarbonate, and UV polymerization of PEGDA. This approach, previously used to produce porous PEG-based hydrogels [33,34,35], offered superior control over hydrogel crosslinking and allowed a notable reduction in polymerization from 30 min [32] to 45 s. 

**Figure 1:**
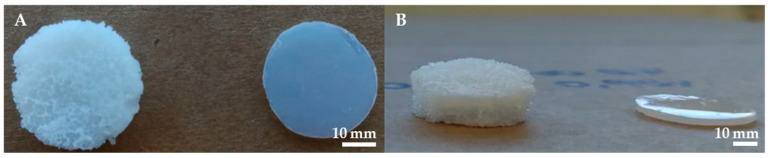
Macroscopic view of a porous PEGDA scaffold produced via gas foaming and UV-based crosslinking and a traditional non-porous PEGDA scaffold. (**A**) Top view. (**B**) Lateral view.

Different formulations of 20% *v*/*v* PEGDA hydrogels were prepared by adjusting the gas foaming and crosslinking parameters to optimize scaffold porosity and pore size. The resulting scaffolds displayed a porous foam structure with a thickness of approximately 6–8 mm, notably higher than the non-porous PEGDA hydrogels produced solely via UV crosslinking, as illustrated in Figure 1.

**Figure 2:**
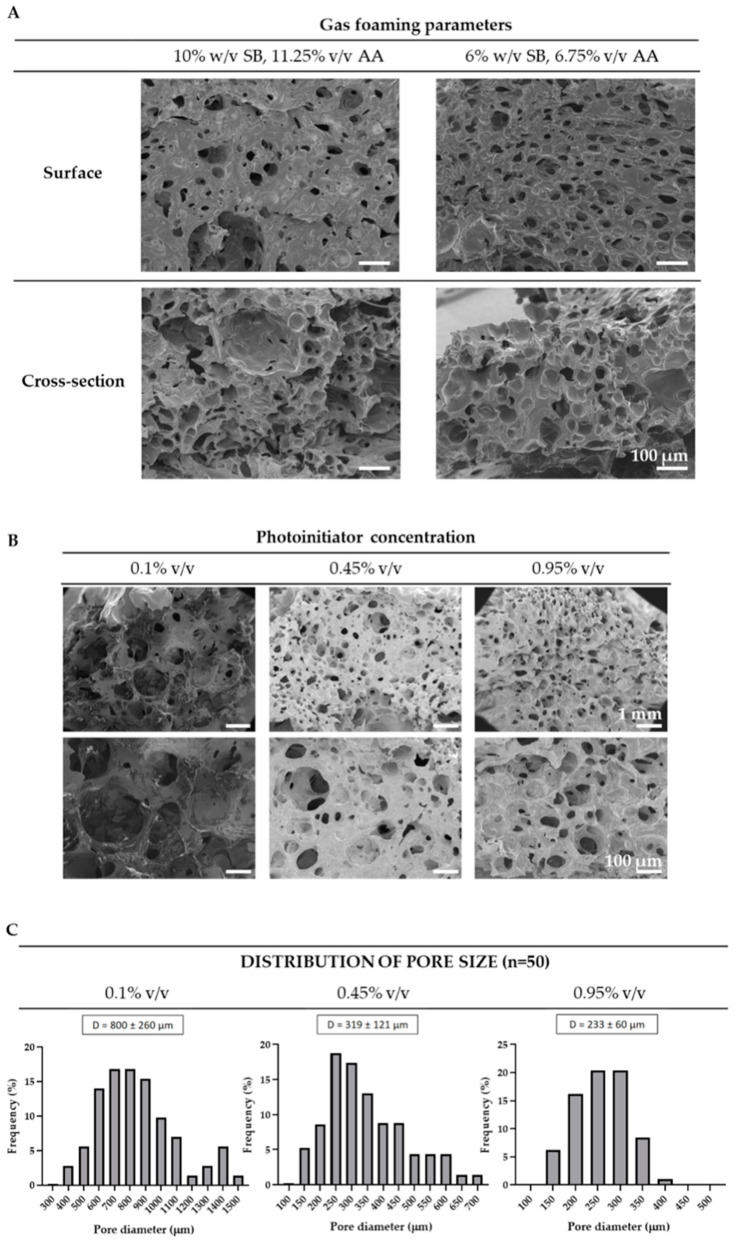
Characterization of porous PEGDA scaffolds. Scanning Electron Microscopy analysis of porous PEGDA scaffolds with different (**A**) gas foaming parameters and (**B**) photoinitiator concentrations. Scale bars: (**A**) 100 µm, (**B**) 1 mm (top) and 100 µm (bottom). (**C**) Size distribution of the pore diameter of scaffolds with different photoinitiator concentrations (*n* = 50).

Initially, we evaluated the effect of the foaming agents’ concentration on scaffold morphology using two formulations based on a prior study by Poursamar et al. [38]: 6% *w*/*v* sodium bicarbonate with 6.75% *v*/*v* acetic acid and 10% *w*/*v* sodium bicarbonate with 11.25% *v*/*v* acetic acid. SEM analysis revealed that increasing the concentration of the foaming agents resulted in thicker and more heterogeneous scaffolds, exhibiting regions lacking pores alongside areas with larger pores, as depicted in Figure 2A. Conversely, the lowest concentrations of sodium bicarbonate and acetic acid yielded scaffolds with smaller and more evenly distributed pores. Hence, the amount of porogen particles and the degree of gas foaming influenced scaffold porosity, as previously evidenced by Lim et al. [39]. Nam et al. reported similar outcomes [40] in PLLA scaffolds fabricated using the gas foaming technique with ammonium bicarbonate. Given the higher homogeneity in pore size, the lowest porogen concentration was selected for further tests. 

Next, we examined how the photoinitiator concentration affected the scaffold pore characteristics, using concentrations of 0.1%, 0.45% and 0.95% (*v*/*v*), respectively. As observed in Figure 2B, all scaffold formulations exhibited spherical-shaped pores evenly dispersed throughout the scaffold thickness. Typically, foam stabilizers such as surfactants are used in gas foaming to prevent liquid drainage and bubble coalescence, promoting the formation of more homogenous foams [41]. However, in this study, foam stabilizers were not used and the porogen (sodium bicarbonate) powder was not incorporated into the prepolymer solution; instead, it was placed at the bottom of each well. This strategic placement was preferred to guarantee that all gas bubbles were generated at the bottom of the well, upon the addition of acetic acid, and subsequently dispersed upwards across the entire scaffold thickness. Pre-mixing the porogen with the prepolymer solution might have resulted in a less uniform pore distribution across the scaffold thickness, potentially requiring the use of foam stabilizers to achieve comparable homogeneity as observed herein. Additionally, higher photoinitiator concentrations resulted in a reduction in the average pore size and pore size distribution, as illustrated in Figure 2C: pore sizes of 800 ± 260 µm, 319 ± 121 µm and 233 ± 60 µm were observed for photoinitiator concentrations of 0.1%, 0.45% and 0.95%, respectively. These results were expected, as increased photoinitiator concentration implies a faster crosslinking, resulting in more stable foams that limit bubble coalescence and the formation of larger pores [31]. 

**Figure 3:**
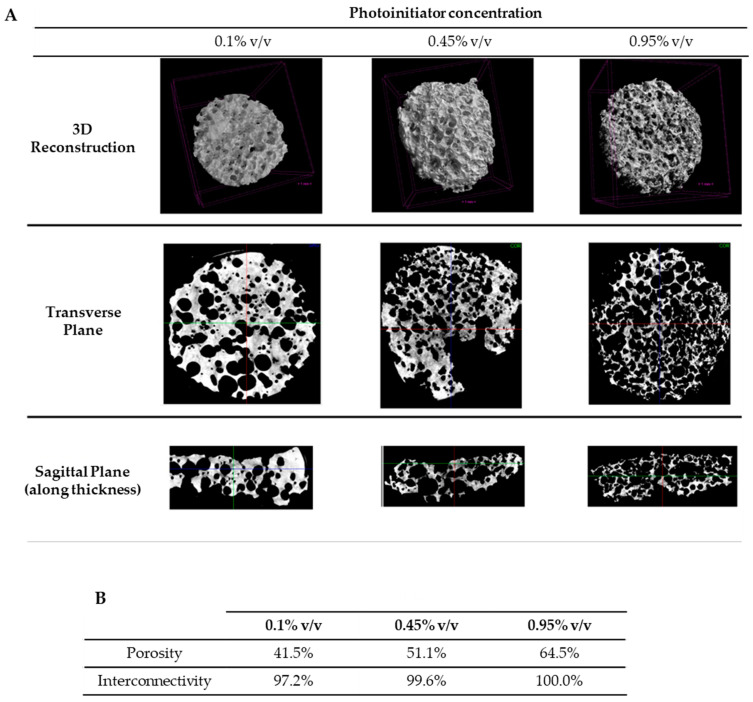
Characterization of porous PEGDA scaffolds with different initiator concentrations (0.1%, 0.45% and 0.95% *v*/*v*). (**A**) μ-CT analysis of PEGDA porous hydrogels. (**B**) Porosity and interconnectivity of the three scaffold formulations.

Micro-computed tomography (μ-CT) analysis corroborated these findings (Figure 3), demonstrating increased scaffold porosity and interconnectivity with higher photoinitiator concentrations. The porosity ranged from 41.5% to 64.5%, consistent with the porosity of porous 20% PEGDA hydrogels reported by Sannino et al. [35] (64.29–68.43%). However, the authors reported smaller pore sizes of 55.6–92.1 µm, likely due to the lower concentration of sodium bicarbonate used (40 mg/mL). For the 0.95% photoinitiator formulation, interconnectivity reached 100%, implying that all pores were accessible for cell seeding, migration and nutrient/oxygen flow [42].

**Figure 4:**
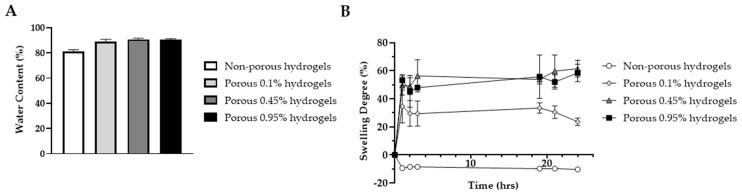
Assessment of 20% *v*/*v* PEGDA hydrogels’ properties. (**A**) Water content and (**B**) swelling behavior of non-porous and porous hydrogels with different photoinitiators concentrations (0.1%, 0.45% and 0.95% *v*/*v*) (*n* = 3).

The water content and swelling degree of the three formulations of porous scaffolds were evaluated alongside non-porous scaffolds with 0.95% photoinitiator concentration. All porous scaffolds exhibited a water content exceeding 89% (Figure 4A), whereas the non-porous scaffold displayed a water content of 81%, in line with the results reported by Nguyen et al. [43] and consistent with the water content reported for cartilage tissue (approximately 80%) [1]. Additionally, water content increased with photoinitiator concentration, albeit not significantly.

The porous hydrogels displayed similar swelling behaviors, reaching swelling degrees of 23.7 ± 3.7%, 61.4 ± 6.0% and 58.4 ± 6.3% after 24 h, for photoinitiator concentrations of 0.1%, 0.45% and 0.95%, respectively (Figure 4B). The swelling degree increased rapidly during the initial 1–3 h and stabilized thereafter, due to the established equilibrium between the ions present in the hydrogel and the surrounding medium [44]. Particularly, the hydrogels with 0.1% photoinitiator concentration had a lower swelling degree compared to those with higher concentrations, possibly due to their reduced thickness and consequently reduced available surface for water uptake. In contrast, the non-porous hydrogels exhibited a distinct swelling behavior, with a swelling degree of approximately −10% throughout the 24 h period, indicating that their polymer network was likely highly compacted and restricted water uptake.

In TE strategies, scaffold pore size plays a pivotal role in facilitating cell penetration, migration, nutrient diffusion and removal of metabolic waste [45]. Several studies have reported differing optimum ranges for cell penetration and chondrogenic differentiation for cartilage TE, depending on factors such as cell type, biomaterial and the chosen architecture. For chondrocytes, scaffold pore sizes of 150–300 µm have been shown to promote cell proliferation and the synthesis of cartilage ECM components, including collagen type II and GAGs [46,47,48]. Conversely, Yamane et al. [49] observed enhanced chondrocyte proliferation and ECM synthesis in scaffolds with pore size of 400 µm, possibly due to differences in scaffold hydrophilicity and biomaterials used. Regarding adipose stem/stromal cells (ASCs), various pore size ranges have been suggested for chondrogenic differentiation, including 100–150 µm [50], 200–300 µm [51] and 350–450 µm [52]. Im et al. [53] reported that scaffolds with pore size of 400 µm promoted enhanced proteoglycan production and expression of cartilage gene markers, while scaffolds with pore size of 200 µm enhanced cell proliferation. Similar findings were reported by Oh et al. [54], who compared pore sizes of 370–400 µm to 90–105 µm. For MSCs, a pore size of 170–500 µm has been identified to support chondrogenesis [55,56]. Within this range, Matsiko et al. [57] found that scaffolds with a pore size of 300 µm enhanced cell proliferation, chondrogenic gene expression and cartilage-like matrix deposition. Considering the broad range of pore sizes deemed suitable for MSC proliferation and chondrogenic differentiation, we chose to seed MSC spheroids in the hydrogel with 0.95% photoinitiator concentration (Section 2.3). This formulation exhibited a pore size range of 170–290 µm and higher interconnectivity in comparison with the other formulations tested.

**Figure 5:**
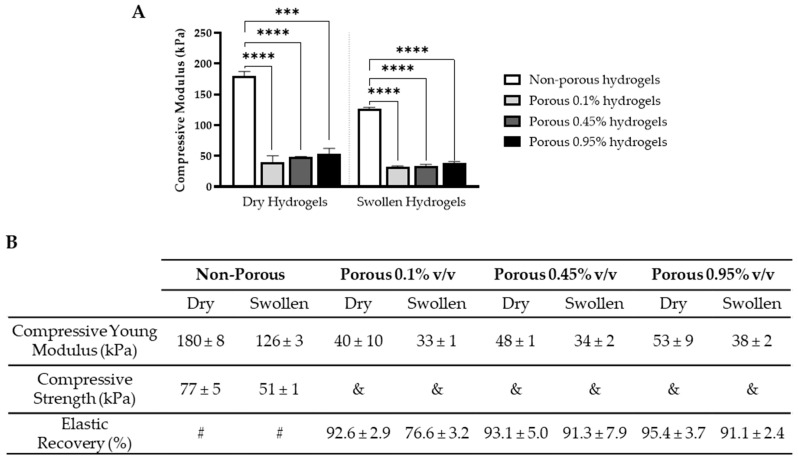
Mechanical properties of porous and non-porous PEGDA scaffolds under uniaxial compression. (**A**) Compressive modulus (kPa) and (**B**) compressive strength (kPa) and elastic recovery (%) of four 20% *v*/*v* PEGDA scaffold formulations, after fabrication and after 1h of swelling: non-porous (0.95% *v*/*v* photoinitiator) and porous scaffolds (0.1, 0.45 and 0.95% *v*/*v* photoinitiator concentrations) (*n* = 3). # Hydrogels broke. & Hydrogels did not break. *** *p* < 0.001; **** *p* < 0.0001.

The mechanical properties, including compressive modulus, strength and elastic recovery, were assessed for both non-porous and porous hydrogels with 0.95% photoinitiator concentration. While all scaffolds exhibited elastomeric behavior (Supplementary Figure S1), porous scaffolds did not break under the applied strain, rendering the evaluation of their compressive strength not feasible. Conversely, non-porous hydrogels presented a compressive strength of 77 ± 5 kPa and a compressive modulus of 180 ± 8 kPa, as shown in Figure 5. These values align with the compressive modulus of PEGDA hydrogels reported by Xiao et al. [58] (183 ± 14 kPa) and Moura et al. [59] (210 ± 20 kPa). However, other studies have reported different compressive modulus for 20% PEGDA hydrogels, including 69–83 kPa [60], 250 kPa [43] and 424–560 kPa [61,62,63], likely due to the different crosslinking mechanisms and the molecular weight of the PEGDA precursor used. The compressive modulus of the porous hydrogels was notably lower compared to their non-porous counterparts, measuring at 40 ± 10 kPa, 48 ± 1 kPa and 53 ± 9 kPa with photoinitiator concentrations of 0.1%, 0.45% and 0.95%, respectively. This was expected since porous hydrogels have a greater void volume compared with non-porous hydrogels due to the presence of macropores, resulting in a reduced effective cross-sectional area, which is key for preserving the original structure under external stress [64,65,66]. Additionally, increasing the crosslinking density of the porous hydrogels resulted in increased mechanical stiffness. This is attributed to the reduced spacing between crosslinks, resulting in a denser and more tightly packed structure that hinders scaffold deformation [67].

Additionally, the porous hydrogels exhibited high elastic recovery of 84.3–99.2%, which indicates their ability to readily return to a shape similar to their original one, making them suitable for use under compressive loading stimuli, as constantly occurs in the articular cartilage within the knee joint. After swelling, both porous and non-porous hydrogels exhibited lower compressive moduli and compressive strength (where applicable). In porous hydrogels, this reduction is directly linked to the water uptake they experience: as the hydrogel swells, its network density decreases, resulting in a softer material [67]. As for the non-porous hydrogels, their decreased compressive modulus after swelling is more likely attributed to their decreased volume fraction, as evidenced by their negative swelling degree, which directly correlates with the elastic modulus [68].

Unlike the non-porous hydrogels, the porous scaffolds exhibited a compressive modulus lower than the reported equilibrium compressive modulus for cartilage (0.08–2.1 MPa) [69]. However, they may offer a more favorable environment for MSC chondrogenic differentiation compared to the non-porous scaffolds. According to Park et al. [70], MSCs seeded on softer matrices (<1 kPa) exhibit increased collagen type II production, in contrast to MSCs seeded on stiffer substrates. This observation has been corroborated by Steward et al. [71] using 3D agarose scaffolds with stiffness ranging from 0.5 kPa to 25 kPa. The authors propose that, although this stiffness range does not fully replicate the values observed for mature human cartilage, it may simulate the cartilage environment during its developmental stage, potentially aiding in MSC differentiation into a cartilage phenotype [72].

### 2.2. In Vitro Biocompatibility of 3D Porous PEGDA Hydrogels with Relevant Cell Populations

**Figure 6:**
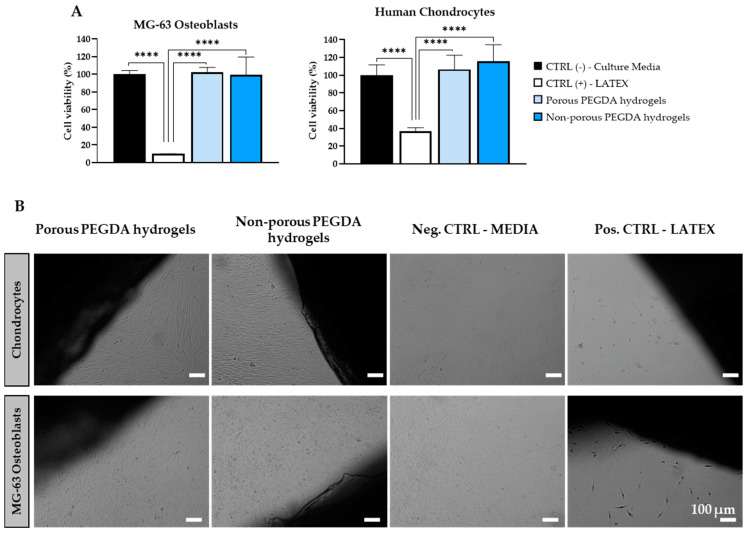
Biocompatibility of non-porous and porous PEGDA scaffolds. (**A**) MTT assay results and (**B**) direct contact test images of porous and non-porous PEGDA scaffolds with human MG-63 osteoblasts and chondrocytes. Scale bar: 100 µm (*n* = 3), **** *p* < 0.0001.

The cytotoxicity of the 3D porous PEGDA hydrogels was assessed through indirect and direct compatibility tests using human MG-63 osteoblastic-like cells and human chondrocytes. The results, depicted in Figure 6, indicate that cells exhibited high viability on both porous and non-porous hydrogels, comparable to the negative control. 

Direct contact tests further supported these findings, showing that both porous and non-porous scaffolds exhibited similar performance to the negative control. MG-63 cells and human chondrocytes remained in confluent monolayers and displayed their characteristic morphology [72,73]. Additionally, no inhibition halo was observed in proximity to the scaffolds, indicating excellent biocompatibility, consistent with previous studies on porous PEGDA scaffolds [74,75].

### 2.3. Chondrogenic Differentiation of MSC Spheroids on 3D Porous PEGDA Hydrogels

**Figure 7:**
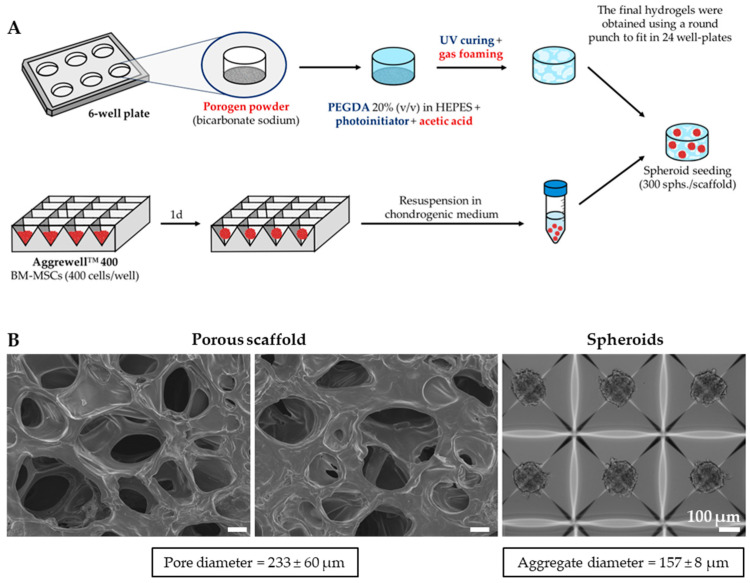
Experimental setup for the development of MSC-spheroid-seeded porous PEGDA hydrogel scaffolds for AC TE. (**A**) Production and seeding of spheroids on porous PEGDA hydrogel scaffolds, fabricated with the gas-foaming technique. (**B**) Pore size of porous PEGDA hydrogels (SEM images, left) and spheroid diameters (brightfield image, right) (*n* = 50). Scale bar: 100 µm.

Spheroids of MSCs, each comprising 400 cells, were generated in AggreWell plates. After 24 h, the spheroids were seeded onto the 3D porous PEGDA hydrogels and cultured in chondrogenic medium for 21 days (Figure 7A). MSCs were employed for their potential to differentiate into chondrocytes and their capacity for extensive in vitro expansion, while the use of spheroids was preferred over single cells to promote cell–cell contact and mimic the cartilaginous condensations characteristic of embryonic development [76]. 

Prior to seeding, the spheroids exhibited a spherical and compact morphology, with an average diameter of 157 ± 8 µm, as illustrated in Figure 7B. Their dimensions closely matched the pore size range of the porous hydrogels, indicating potential for effective colonization of the construct. Additionally, it has been reported that aggregates of similar size exhibit superior chondrogenic differentiation compared to 1–2 mm pellets [77].

**Figure 8:**
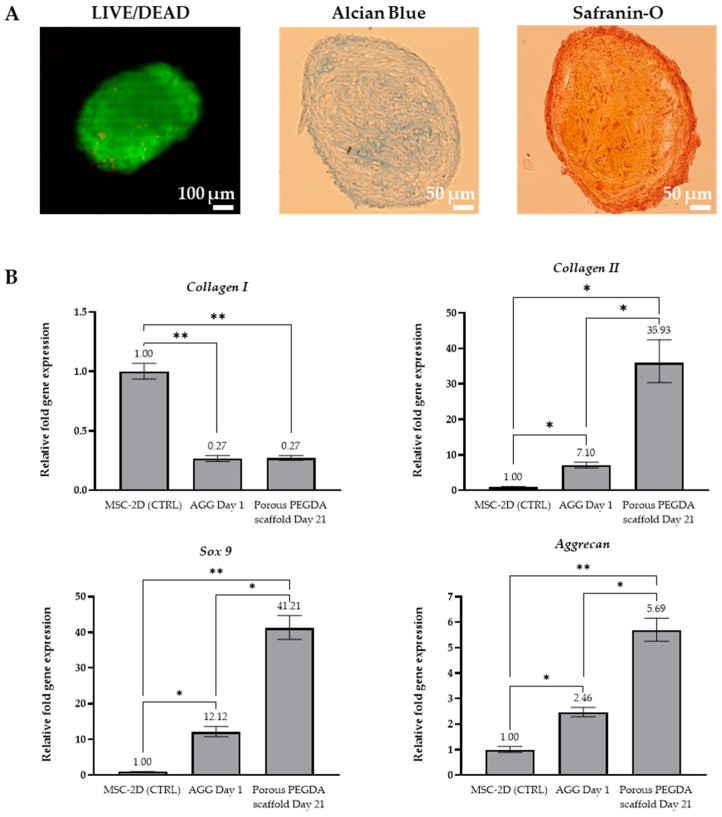
Evaluation of chondrogenic differentiation of MSC spheroids seeded in porous PEGDA scaffolds. (**A**) Live/Dead (green: live cells, red: dead cells), Alcian Blue and Safranin-O stainings of MSC spheroids at day 21 post-seeding. (**B**) qRT-PCR analysis of *COL1A1*, *COL2A1*, *SOX9* and *ACAN* in 2D cultures (control), 3D spheroids (day 1) and in 3D spheroids cultured on PEGDA porous hydrogels (day 21 post-seeding). Gene expression levels were normalized to that of glyceraldehyde 3-phosphate dehydrogenase (*GAPDH,* endogenous control) and calculated as fold-change relative to the control sample (2D MSC cultures at day 0, before spheroid formation and scaffold seeding). (*n* = 3), * *p* < 0.05, ** *p* < 0.01.

After 21 days of culture, Safranin-O stainings showed that the MSC-derived spheroids were localized within the pores of the scaffold (Supplementary Figure S2). The aggregates retained their initial spherical morphology and most cells within the aggregate remained viable, as shown in Figure 8A. Although a small number of dead cells were observed, no necrotic center was evident, largely due to the small diameter of the aggregates, which allowed sufficient nutrient and oxygen diffusion. Additionally, the metabolic activity of the aggregates was maintained throughout the whole culture period, exhibiting a non-significant increase between day 1 and day 21 (Supplementary Figure S3). Alcian Blue and Safranin-O stainings performed on the final spheroids further revealed the presence of proteoglycans and GAGs, respectively, both typical components of the articular cartilage ECM.

To assess the expression of chondrogenic markers, quantitative real-time polymerase chain reaction (qRT-PCR) was performed on MSC aggregates at day 1 (before differentiation) and on spheroid-seeded PEGDA porous hydrogels at day 21 (after differentiation). Specifically, the expression levels of *COL1A1* (collagen type I, a marker of bone tissue), *COL2A1* (collagen type II, indicative of cartilaginous tissue), *SOX9* (a transcription factor for chondrogenic genes) and *ACAN* (aggrecan, main cartilage proteoglycan) were evaluated and normalized to 2D MSC cultures at day 0 (before spheroid formation and scaffold seeding). As illustrated in Figure 8B, MSC aggregates exhibited decreased expression of *COL1A1* (0.27-fold) and increased expression of *COL2A1* (7.1-fold), *SOX9* (12.1-fold) and *ACAN* (2.5-fold), compared to 2D MSC cultures, which is consistent with the literature [76,78]. This suggests that, while MSC aggregates undergo some level of pre-conditioning, their 3D configuration alone is insufficient to initiate chondrogenesis. Chondrogenic stimuli, such as medium supplemented with TGF-βs, are still required to support proper MSC chondrogenesis [76].

In spheroid-seeded PEGDA hydrogels, following 21 days of differentiation, a similar trend of *COL1A1* downregulation and *COL2A1*, SOX9 and *ACAN* upregulation was observed, when compared to 2D culture, confirming successful chondrogenic differentiation. Notably, there was a significant increase in the expression of *COL2A1* (35.9-fold) and SOX9 (41.2-fold), while *ACAN* levels exhibited a more moderate increase (5.7-fold). In comparison with MSC aggregates at day 1, the aggregates cultured in PEGDA porous hydrogels showed similar expression of *COL1A1* but increased expression of *COL2A1* (5.1-fold), *SOX9* (3.4-fold) and *ACAN* (2.3-fold). These findings not only highlight the capacity of the 3D porous PEGDA hydrogels to support chondrogenesis but also demonstrate that combining MSC aggregates with a porous PEGDA scaffolds of suitable pore size might be a more effective approach for inducing chondrogenic differentiation, when compared to scaffold-free MSC aggregates and conventional 2D monolayer cultures. Hence, the spheroid-seeded 3D porous PEGDA hydrogel scaffolds hold significant promise for cartilage TE or in vitro OA modelling strategies.

## 3. Conclusions

In summary, 3D porous PEGDA hydrogels were developed using the gas foaming technique coupled with UV-induced crosslinking and presented different porosities according to the foaming agents and photoinitiator concentrations used. The optimal porosity for cartilage TE and MSC chondrogenic differentiation was obtained using the 6% sodium bicarbonate, 6.75% acetic acid and 0.95% photoinitiator formulation, yielding pore sizes of 170–290 µm. Moreover, this formulation showed high cytocompatibility both with human chondrocytes and osteoblast-like cells. While the scaffold presented a compressive modulus of 53 ± 9 kPa, lower than the stiffness range of mature cartilage (0.08–2.1 MPa) [69], it may mimic more closely the stiffness of cartilage during its developmental stage. Consequently, the porous hydrogel scaffold could potentially facilitate MSC chondrogenic differentiation more effectively than non-porous PEGDA hydrogels of higher compressive modulus. After being cultured on the porous hydrogels in chondrogenic medium, MSC-derived aggregates of 157 ± 8 µm in diameter were found within the scaffold’s pores, exhibiting increased expression of *COL2A1*, *SOX9* and *ACAN*, and decreased expression of *COL1A1* in comparison to 2D MSC cultures before differentiation and scaffold-free MSC aggregates, which exhibited a similar trend, but showed less pronounced upregulation of *COL2A1*, *SOX9* and *ACAN* genes. This suggests that, while MSC aggregates experienced a certain degree of pre-conditioning due to their 3D configuration, chondrogenesis was significantly enhanced in the 3D porous PEGDA hydrogel scaffolds after differentiation, highlighting its suitability for cartilage TE strategies or in vitro disease modelling.

## 4. Materials and Methods

### 4.1. Materials

Poly(ethylene glycol) diacrylate (PEGDA, 575 Da), HEPES, phosphate buffer saline (PBS), sodium bicarbonate, acetic acid, glycine, isopropanol, hydrochloric acid (HCl), paraformaldehyde (PFA), the photoinitiator dimethoxy-2-phenylacetophenone (DMPA), N-vinyl-pyrrolidone (NVP), 1X MEM non-essential amino acids, L-ascorbic acid, ascorbic acid 2-phosphate, L-Proline, dexamethasone, Alcian Blue 8GX powder, Safranin-O dye and the 3-(4,5-Dimethylthiazol-2-yl)-2,5-Diphenyltetrazolium Bromide) (MTT) assay kit were acquired from Sigma-Aldrich (St. Louis, MI, USA). Dulbecco’s Modified Eagle Medium (DMEM), high-glucose DMEM, fetal bovine serum (FBS), antibiotic-antimycotic mixture (Anti-Anti), sodium pyruvate, Collagenase Type IV powder, High-capacity cDNA reverse transcription kits, MicroAmp^TM^ Fast Optical 96-well reaction PCR plates, SYBR^TM^ Green Master Mix, LIVE/DEAD^TM^ viability/cytotoxicity kit and AlamarBlue^TM^ Cell Viability Reagent were supplied by Thermo Fisher Scientific (Waltham, MA, USA). The RNeasy Mini Kit for RNA extraction was obtained from Qiagen (Hilden, Germany). ITS^TM^+ Premix supplement (6.25 μg/mL bovine insulin; 6.25 μg/mL transferrin; 6.25 μg/mL selenous acid; 5.33 μg/mL linoleic acid; 1.25 μg/mL bovine serum albumin) and ultra-low attachment 24-well culture plates were purchased from Corning Inc. (New York, NY, USA). Transforming growth factor-β3 (TGF-β3) was obtained from R&D Systems (Minneapolis, MN, USA). AgreeWell^TM^400 microwell plates were acquired from STEMCELL Technologies (Vancouver, BC, Canada).

### 4.2. Fabrication of 3D Porous PEGDA Hydrogels

For the production of 3D porous PEGDA hydrogels, the gas foaming technique was combined with UV light-based crosslinking. PEGDA (MW 575 Da) was dissolved in a 10 mM HEPES (238 g/mol) solution in 0.01M PBS to achieve a final concentration of 20% *v*/*v*. To prepare the photoinitiator solution, 300 mg of DMPA were added to each mL of N-vinyl-pyrrolidone (NVP) and the solution was subjected to vigorous agitation in a thermomixer to allow complete dissolution of the powder before use and was kept in the dark. After the addition of the photoinitiator, the final solution was filtered through a 0.22 μm filter (Merck Millipore, Burlington, MA, USA). To optimize the porosity/pore size and physical features of the porous hydrogels, different formulations were tested as described in Table 1. To fabricate the scaffolds, the sodium bicarbonate powder was evenly spread on the bottom of a well of a 6-well culture plate. Afterwards, 1.8 mL of PEGDA (with photoinitiator) solution and the proportional amount of acetic acid were poured into the well and the plate was exposed to UV light for 45 s. The reaction occurring between acetic acid and sodium bicarbonate leads to the formation of carbon dioxide bubbles, which are responsible for the generation of pores within the hydrogel structure. Non-porous hydrogels were produced using the described protocol (without sodium bicarbonate). The final hydrogel scaffolds were obtained using a round punch (with a diameter of 14 mm) in order to perfectly fit the size of a well of a 24-well culture plate.

### 4.3. Characterization of 3D Porous PEGDA Hydrogels

#### 4.3.1. Scanning Electron Microscopy (SEM) and Pore Size Measurements

The morphology and pore size of the produced hydrogels were observed by Scanning Electron Microscopy (SEM) analysis. Before the imaging, the samples were coated with an Au/Pd layer of 30 nm using a Polaron model E5100 coater (Quorum Technologies, Lewes, UK). Images were obtained using a Field Emission Gun Scanning Electron Microscope (FEG-SEM) equipment (JEOL, JSM-7001F model, Tokyo, Japan) with the accelerated voltage set at 15 kV. Pore size measurements were performed using the ImageJ software version 1.53t (NIH, Bethesda, MD, USA). The average pore sizes were calculated from 50 (*n* = 50) well-defined individual pores from at least three different SEM images. 

#### 4.3.2. Micro-Computed Tomography (μ-CT) Analysis

The internal microstructure of the different porous PEGDA hydrogels was obtained through μ-CT analysis using a SkyScan 1174v2 version 1.1 (Bruker, Billerica, MA, USA) scanner equipment. The acquisition was made using the following parameters: image pixel size of 16.65 μm; source voltage of 50 kV; source current of 800 μA; exposure time of 700 ms; rotation step of 0.7° (no filter). Three-dimensional reconstruction was carried out using the Software NRecon version 1.7.4.6 (Bruker) and the CTVox version 3.3.1 (Bruker) program was used to provide a 3D visualization of the scanned samples. μ-CT analysis allows the evaluation of scaffold microstructural features such as porosity and interconnectivity, which are calculated as:

(1) $$Porosity\;\left(\%\right)=\frac{V_{pores}}{V_{pores}+V_{scaffold}}\times 100$$

(2) $$Interconnectivity\;\left(\%\right)=\frac{V_{open\;pores}}{V_{open\;pores}+V_{closed\;pores}}\times 100$$

#### 4.3.3. Swelling Behavior and Water Content 

A swelling test was performed on 20% (*v*/*v*) 3D porous (three formulations with different concentrations of photoinitiator—0.1%, 0.45%, 0.95%) and non-porous hydrogels. Immediately after the crosslinking process, the hydrogels were extracted from the mold, weighed (*W*_0_) and left to soak in PBS. At each time point, the samples were removed and weighed (*W_s_*, especially care was taken to remove the excess PBS from the surface of the hydrogels, so that only the weight of incorporated PBS was taken into account). The swelling equilibrium weight (*W_s_*) and the dry weight (*W*_0_) were then used to calculate the % of swelling ratio:

(3) $$Swelling\;ratio\;\left(\%\right)=\frac{\left(W_{s}-W_{0}\right)}{W_{0}}\times 100$$

To measure the water content, the hydrogels were dried for 24 h at 37 °C after fabrication and weighed (*W_d_*). The water content was calculated considering the initial (*W_0_*) and dried (*W_d_*) weights of the hydrogels:

(4) $$Water\;content\;\left(\%\right)=\frac{\left(W_{0}-W_{d}\right)}{W_{0}}\times 100$$

Three independent hydrogels (*n* = 3) were considered for both swelling ratio and water content analyses.

#### 4.3.4. Mechanical Properties under Compressive Testing

The compressive tests of the hydrogel samples (diameter of 14 mm and height of 4 mm, *n* = 3 samples per condition) were performed on an Univert mechanical tester (Model UV-200-01; CellScale Biomaterials Testing, Waterloo, ON, Canada) equipped with a 10 N load cell and using a compression rate of 1 mm/min. The obtained force–displacement curves were then transformed into stress–strain plots given the sample dimensions and considering Equations (5) and (6).

(5) $$Stress=\sigma =\frac{F}{A}$$

where *F* (Newton, N) is the applied force and *A* (mm^2^) is the cross-section area.

(6) $$Strain=\varepsilon =\frac{\Delta L}{L_{0}}$$

where Δ*L* (mm) is the displacement and *L_0_* (mm) is the initial height.

The compressive Young’s modulus (Equation (7)) can be calculated from the initial linear strain region (0–15%) of the stress–strain curve.

(7) $$Young’s\;Modulus=\frac{\sigma }{\varepsilon }$$

where *σ* (N·mm^−2^ = MPa) is the stress and *ε* (non-dimensional) is the strain.

### 4.4. In Vitro Cytotoxicity Tests

The cytocompatibility of the hydrogels (diameter: 14 mm and height: 3 mm) was assessed using human chondrocytes and human MG-63 osteoblast-like cells in conformity with the ISO 10993-5 guidelines [79]. Human chondrocytes were acquired from CELL Applications, Inc. (San Diego, CA, USA) and cultured using a medium composed of high-glucose DMEM supplemented with 10% FBS (*v*/*v*), 1× MEM non-essential amino acids, 0.4 mM L-Proline, 0.2 mM L-Ascorbic acid and 1% (*v*/*v*) Anti-anti. Human MG-63 osteoblast-like cells were obtained from ATCC (CRL-1427™, Manassas, VA, USA) and cultured in DMEM supplemented with 10% FBS and 1% Anti-anti. Both cell types were cultured in an incubator at 37 °C and 5% CO_2_ in a humidified atmosphere and passaged to new flasks when confluence (≈80–90%) was reached. The culture medium was completely renewed every 2–3 days. Porous and non-porous 20% (*v*/*v*) PEGDA hydrogels were sterilized by several washes and incubation overnight in a 1% Anti-anti solution (in PBS). The samples were evaluated by means of indirect (extracts) and direct contact cytotoxicity tests. For both cell types, the cells were seeded on tissue-culture-treated polystyrene plates at a density of 1 *×* 10^5^ (24-well plate) and 2 × 10^5^ (12-well plate) per well for the indirect and direct contact tests, respectively. The cells were then cultured for 24 h at 37 °C and 5% CO_2_ to achieve confluent monolayers. Cells cultured in the respective culture media were the negative controls, while latex material was used as the positive control of a cytotoxic response. To prepare the extraction media for the indirect tests, the hydrogels were incubated in culture medium (ratio of 0.2 g/mL) for 24 h at 37 °C and 5% CO_2_. Afterwards, the cells were cultured in the respective extraction medium for 72 h at 37 °C and 5% CO_2_ in a humidified incubator. When the incubation period was finished, the extraction media were removed and the MTT-based in vitro toxicology assay kit was used according to the manufacturer’s instructions. Briefly, the cell cultures were incubated in a 1 mg/mL MTT solution (prepared in PBS, yellow color) for 4h at 37 °C. Then, the violet formazan product, resulting from the reduction of MTT by metabolically viable cells, was dissolved with the appropriate MTT solvent (0.1 N HCl in anhydrous isopropanol) under agitation for 5 min. Finally, the resultant solutions were transferred to a 96-well plate and the absorbance values were measured using a microplate reader (Infinite 200 PRO, Tecan, Männedorf, Switzerland) at 570 nm. The percentage of cell viability of the different samples was calculated by comparison with the values obtained for the negative control cultures, which were considered to have 100% viable cells. Four independent samples (*n* = 4) of each condition were assayed, and the absorbance of each sample was read in triplicate. 

Regarding the direct contact assays, each hydrogel condition (*n* = 3, porous vs. non-porous) and respective controls were placed on top of a confluent monolayer of chondrocytes or osteoblasts and incubated for 72 h at 37 °C in a humidified atmosphere with 5% CO_2_. The viability and morphology of the cells in direct contact with the materials were qualitatively assessed using an inverted optical microscope (LEICA DMI3000B, Leica Microsystems, Wetzlar, Germany) connected to a digital camera (Nikon DXM1200F, Nikon Instruments Inc., Tokyo, Japan).

### 4.5. MSC Spheroids Culture and Differentiation on 3D Porous PEGDA Hydrogels

hBM-MSCs (36 years healthy male donor) were isolated from bone marrow aspirates according to protocols previously established at the Institute for Bioengineering and Biosciences (iBB)—Instituto Superior Técnico (IST), Lisbon, Portugal [20]. Bone marrow aspirates were obtained from Instituto Português de Oncologia Francisco Gentil, Lisboa, Portugal, after written informed consent and with the approval of the Ethics Committee of the respective clinical institution. All human samples were obtained from healthy donors after written informed consent according to Directive 2004/23/EC of the European Parliament and of the Council of 31 March 2004, on setting standards of quality and safety for the donation, procurement, testing, processing, preservation, storage and distribution of human tissues and cells (Portuguese Law 22/2007, 29 June). hBM-MSCs were thawed from a frozen stock (nitrogen liquid tanks) and cultured in DMEM supplemented with 10% FBS (MSC qualified) and 1% Anti-anti at 37 °C and 5% CO_2_. The culture medium was fully changed every 2–3 days and the cells were passaged when a confluence of around 80–90% was reached. All experiments were carried out using hBM-MSCs in passages between 4 and 6.

Prior to the preparation of the spheroids, 1 mL of AggreWell Rinsing solution (STEMCELL Technologies, Vancouver, BC, Canada) was added to each well of the AggreWell^TM^400 plate to minimize the adhesion of the cells to the microwells. Then, the plate was centrifuged to remove the rinsing solution, 500 μL of culture media were added to each well and the plate was centrifuged again to completely eliminate any air bubbles. To produce the spheroids in accordance with the manufacturer’s protocol, hBM-MSCs were harvested, centrifuged and resuspended in an appropriate media volume to achieve spheroids of 400 cells and considering that each well of an AggreWell plate generates 1200 spheroids. Thus, 500 μL of the cell suspension were added to each well, followed by an incubation of 5 min at room temperature and a centrifugation (1500 rpm for 10 min) of the AggreWell plate. After 18 h of incubation at 37 °C and 5% CO_2_, the samples were imaged to assess the size of the spheroids produced. The area of the spheroids (*n* = 50) was measured using the area measurement algorithm from ImageJ software version 1.53t (National Institutes of Health, USA) after adjusting the threshold up to the border of the spheroid. For each well, the spheroids were carefully transferred to a falcon tube, left to deposit on the bottom, and the DMEM + 10% FBS (MSC) media was removed. The 1200 spheroids (one well) were resuspended in 200 μL of chondrogenic medium consisting of high-glucose DMEM supplemented with 100 nM dexamethasone, 50 μg/mL ascorbic acid 2-phosphate, 40 μg/mL L-Proline, 1mM sodium pyruvate, ITS™+ Premix, 1% Anti-anti and 10 ng/mL TGF-β3. Upon gentle mixing, a 50 μL suspension (containing around 300 spheroids) was placed dropwise on different spots on the top of each hydrogel scaffold placed in ultra-low attachment plates. The constructs were then incubated at 37 °C and 5% CO_2_ for 1 h before adding 1 mL of chondrogenic medium to each well. The cultures were maintained for 21 days under chondrogenic induction conditions at 37 °C and 5% CO_2_, and the media was carefully changed twice a week. According to previously obtained results for hydrogel porosity, pore size and biocompatibility, the formulation selected for the spheroid differentiation studies was 20% *v*/*v* PEGDA, 0.95% *v*/*v* photoinitiator DMPA, 6% *w*/*v* Sodium bicarbonate, 6.75% *v*/*v* Acetic acid.

### 4.6. Assessment of Spheroid Viability and Chondrogenic Differentiation after Culture on 3D Porous PEGDA Hydrogels

#### 4.6.1. Live/Dead Staining and Alamar Blue Assay

The viability of the spheroids cultured on the 3D porous hydrogels for 21 days under chondrogenic induction was assessed using the LIVE/DEAD assay kit. Briefly, the spheroids were collected from the porous hydrogels, placed in a new plate, and incubated with a solution of 4 μM calcein-AM and 2 μM ethidium bromide (prepared in PBS) for 1 h at room temperature and protected from light. Then, the spheroids were washed once with PBS and immediately imaged using a LEICA DMIB3000B inverted fluorescence microscope. The metabolic activity of the MSC-derived spheroids on the porous hydrogels was monitored throughout the culture period at days 1, 2, 7, 14 and 21 using the AlamarBlue^TM^ assay following the manufacturer’s guidelines. Briefly, a 10% (*v*/*v*) AlamarBlue solution in cell culture medium was added to the MSC spheroid-seeded hydrogels and incubated for 3 h at 37 °C, 5% CO_2_. Fluorescence intensity was measured in a microplate reader (Infinite 200 PRO, Tecan, Männedorf, Switzerland) at an excitation/emission wavelength of 560/590 nm. The results are presented as the fluorescence intensity ratio between the spheroid-seeded hydrogels and acellular hydrogels (blank controls) at the respective time points. Three (*n* = 3) independent samples were used in the analysis.

#### 4.6.2. Alcian Blue and Safranin-O Stainings

The spheroids harvested from the porous PEGDA hydrogels (and also one sample of spheroid-containing porous hydrogels) were fixed in 4% (*v*/*v*) PFA solution (in PBS) for 20 min at room temperature, washed twice with PBS and incubated overnight in 15% (*v*/*v*) sucrose solution at 4 °C. The samples were embedded in 7.5%/15% (*v*/*v*) gelatin/sucrose solution and frozen in isopentane at −80 °C. Sections measuring 12 μm were cut on a cryostat-microtome (Leica CM3050S, Leica Microsystems, Wetzlar, Germany), collected on Superfrost^TM^ Microscopic Slides (Thermo Fisher Scientific) and stored at −20 °C.

To test for the presence of sulfated glycosaminoglycans (GAGs), typical constituents of articular cartilage ECM, the histological sections were stained with Alcian Blue and Safranin-O. The sections were first de-gelatinized in PBS for 45 min at 37 °C and incubated in a 0.1 M Glycine solution for 10 min at room temperature. The spheroids were then stained with a 1% (*w*/*v*) Alcian Blue solution (prepared in 0.1 N HCl) for 1 h at room temperature. Alternatively, other samples were stained with a 0.1% (*w*/*v*) Safranin-O solution (in distilled water) and left incubating for 20 min at room temperature. For both stainings, the slides were washed several times in PBS and imaged in an inverted optical microscope LEICA DMI3000B equipped with a Nikon DXM1200F digital camera.

#### 4.6.3. Quantitative Real-Time Polymerase Chain Reaction (qRT-PCR) Analysis

To assess the chondrogenic differentiation of the spheroids, a qRT-PCR assay was performed considering three different conditions: 2D cultured cells at Day 0; aggregates removed from the AggreWell plate at Day 1; and aggregates cultured on the porous PEGDA hydrogels for 21 Days in chondrogenic medium.

Prior to the RNA extraction, the spheroids were treated with a 0.3% *w*/*v* Collagenase IV solution for 3 h at 37 °C to allow cell disaggregation and lysis. RNA was isolated using the RNeasy Mini Kit following the manufacturer’s guidelines and quantified in a NanoVue Plus^TM^ spectrophotometer (GE Healthcare, Chicago, IL, USA). Afterwards, cDNA was synthesized from the purified RNA using High-Capacity cDNA Reverse Transcription Kit (Applied Biosystems^TM^, Thermo Fisher Scientific, Waltham, MA, USA) according to the manufacturer’s protocol. Briefly, previously prepared reaction mixtures were incubated in a T100™ thermal cycler (Bio-Rad, Hercules, CA, USA) for 10 min at 25 °C, 120 min at 37 °C and 5 min at 85 °C, and then were kept at 4 °C until further use. qRT-PCR analysis was performed using SYBR^TM^ Green Master Mix and StepOnePlus real-time PCR system (Applied Biosystems^TM^, Thermo Fisher Scientific). All reactions were carried out at 95 °C for 10 min, followed by 40 cycles of 95 °C for 15 s and 60 °C for 1 min. All samples were analyzed in triplicates (*n* = 3). The CT values obtained were normalized against the expression of the housekeeping gene glyceraldehyde-3-phosphate (GADPH), and their analysis was performed using the 2^−ΔΔCT^ method. Gene expression results for the target genes *COL1A1*, *COL2A1*, *SOX9* and *ACAN* were determined as a fold-change relative to the baseline expression of the target genes in the control sample (2D hBM-MSC cultures before scaffold seeding). The primer sequences used in the qRT-PCR analysis are presented in Table 2.

### 4.7. Statistical Analysis

Results are presented as average values ± standard deviation (SD). In this study, all the experimental procedures were performed using three independent samples (*n* = 3), unless specified differently. Statistical analysis of the results was performed through one-way ANOVA, followed by Tukey post hoc test using the GraphPad Prism software version 8.4.2. Data were considered statistically significant when the *p*-values obtained were lower than 0.05 (95% confidence intervals, * *p* < 0.05).

## Figures and Tables

**Table 1:** Summary of the formulations tested to produce porous PEGDA hydrogels.

PEGDA concentration	20% *v*/*v* PEGDA in 10 mM HEPES solution
Photoinitiator concentration	0.1% *v*/*v* 0.45% *v*/*v* 0.95% *v*/*v*
UV curing time	45 s
Gas foaming parameters	6% *w*/*v* Sodium bicarbonate + 6.75% *v*/*v* Acetic acid 10% *w*/*v* Sodium bicarbonate + 11.25% *v*/*v* Acetic acid

**Table 2:** Primer sequences (forward and reverse) used in the qRT-PCR analysis.

Gene	Forward Primer Sequence	Reverse Primer Sequence
*GADPH*	5′-GGTCACCAGGGCTGCTTTTA-3′	5′-CCTGGAAGATGGTGATGGGA-3′
*COL1A1*	5′-CATCTCCCCTTCGTTTTTGA-3′	5′-CCAAATCCGATGTTTCTGCT-3′
*COL2A1*	5′-GGAATTCCTGGAGCCAAAGG-3′	5′-AGGACCAGTTCTTGAG-3′
*SOX9*	5′-TACGACTACACCGACCACCA-3′	5′-TTAGGATCATCTCGCCCATC-3′
*ACAN*	5′-CACTGGCGAGCACTGTAACAT-3′	5′-TCCACTGGTAGTCTTGGGCAT-3′

## Data Availability

All data from this study are available from the authors upon request.

## Supplementary Materials

The following supporting information can be downloaded at: https://www.mdpi.com/article/10.3390/gels10070422/s1. Figure S1: Representative Stress–Strain curve for an unconfined compression test for three conditions of non-porous hydrogels with 0.95% *v*/*v* photoinitiator and porous hydrogels with 0.1%, 0.45% or 0.95% *v*/*v* photoinitiator concentration, after fabrication and after swelling for 24 h; Figure S2: Safranin-O staining on a histological section of the 3D porous PEGDA scaffold seeded with MSC aggregates; Figure S3: Alamar Blue assay results for MSC aggregates cultured on PEGDA porous hydrogel scaffolds at days 1, 2, 7, 14 and 21.

## References

[1] Fox A.J.S., Bedi A., Rodeo S.A. (2009). The basic science of articular cartilage: Structure, composition, and function. Sports Health.

[2] Kempson G.E., Sokoloff L. (1980). The mechanical properties of articular cartilage. The Joints and Synovial Fluid.

[3] Roseti L., Desando G., Cavallo C., Petretta M., Grigolo B. (2019). Articular cartilage regeneration in osteoarthritis. Cells.

[4] Buckwalter J.A., Mankin H.J., Grodzinsky A.J. (2005). Articular cartilage and osteoarthritis. Instr. Course Lect..

[5] Armiento A.R., Stoddart M.J., Alini M., Eglin D. (2018). Biomaterials for articular cartilage tissue engineering: Learning from biology. Acta Biomater..

[6] Kwon H., Paschos N.K., Hu J.C., Athanasiou K. (2016). Articular cartilage tissue engineering: The role of signaling molecules. Cell Mol. Life Sci..

[7] Athanasiou K.A., Darling E.M., Hu J.C., Seidel A. (2010). In vitro tissue engineering of hyaline articular cartilage. Articular Cartilage Tissue Engineering.

[8] Flik K.R., Lewis P., Kang R.W., Cole B.J. (2006). Articular cartilage. Clinical Sports Medicine.

[9] GBD 2021 Osteoarthritis Collaborators (2023). Global, regional, and national burden of osteoarthritis, 1990–2020 and projections to 2050: A systematic analysis for the global burden of disease study 2021. Lancet Rheumatol..

[10] Gu Y.-T., Chen J., Meng Z.-L., Ge W.-Y., Bian Y.-Y., Cheng S.-W., Xing C.-K., Yao J.-L., Fu J., Peng L. (2017). Research progress on osteoarthritis treatment mechanisms. Biomed. Pharmacother..

[11] World Health Organization Osteoarthritis. https://www.who.int/news-room/fact-sheets/detail/osteoarthritis.

[12] Allen K.D., Thoma L.M., Golightly Y.M. (2022). Epidemiology of osteoarthritis. Osteoarthr. Cartil..

[13] Plotnikoff R., Karunamuni N., Lytvyak E., Penfold C., Schopflocher D., Imayama I., Johnson S.T., Raine K. (2015). Osteoarthritis prevalence and modifiable factors: A population study. BMC Public Health.

[14] Borrelli J., Olson S.A., Godbout C., Schemitsch E.H., Stannard J.P., Giannoudis P.V. (2019). Understanding articular cartilage injury and potential treatments. J. Orthop. Trauma.

[15] Wei W., Dai H. (2021). Articular cartilage and osteochondral tissue engineering techniques: Recent advances and challenges. Bioact. Mater..

[16] Zhang Z., Mu Y., Zhou H., Yao H., Wang D.-A. (2023). Cartilage tissue engineering in practice: Preclinical trials, clinical applications, and prospects. Tissue Eng. Part B Rev..

[17] Silva J.C., Udangawa R.N., Chen J., Mancinelli C.D., Garrudo F.F.F., Mikael P.E., Cabral J.M.S., Ferreira F.C., Linhardt R.J. (2023). Kartogenin-loaded coaxial PGS/PCL aligned nanofibers for cartilage tissue engineering. Mater. Sci. Eng. C Mater. Biol. Appl..

[18] Huang Y.-Z., Xie H.-Q., Silini A., Parolini O., Zhang Y., Deng L., Huang Y.-C. (2017). Mesenchymal stem/progenitor cells derived from articular cartilage, synovial membrane and synovial fluid for cartilage regeneration: Current status and future perspectives. Stem Cell Rev..

[19] Tan A.R., Hung C.T. (2017). Concise review: Mesenchymal stem cells for functional cartilage tissue engineering: Taking cues from chondrocyte-based constructs. Stem Cells Transl. Med..

[20] Silva J.C., Han X., Silva T.P., Xia K., Mikael P.E., Cabral J.M.S., Ferreira F.C., Linhardt R.J. (2020). Glycosaminoglycan remodeling during chondrogenic differentiation of human bone marrow-/synovial-derived mesenchymal stem/stromal cells under normoxia and hypoxia. Glycoconj. J..

[21] Annabi N., Nichol J.W., Zhong X., Ji C., Koshy S., Khademhosseini A., Dehghani F. (2010). Controlling the porosity and microarchitecture of hydrogels for tissue engineering. Tissue Eng. Part B Rev..

[22] Gäbler S., Stampfl J., Koch T., Seidler S., Schüller G., Redl H., Juras V., Trattnig S., Weidisch R. (2009). Determination of the viscoelastic properties of hydrogels based on polyethylene glycol diacrylate (PEG-DA) and human articular cartilage. Int. J. Mater. Eng. Innov..

[23] Williams C.G., Kim T.K., Taboas A., Malik A., Manson P., Elisseeff J. (2003). In vitro chondrogenesis of bone marrow-derived mesenchymal stem cells in a photopolymerizing hydrogel. Tissue Eng..

[24] Moon J.J., Hahn M.S., Kim I., Nsiah B.A., West J.L. (2009). Micropatterning of poly(ethylene glycol) diacrylate hydrogels with biomolecules to regulate and guide endothelial morphogenesis. Tissue Eng. Part A.

[25] Yang F., Williams C.G., Wang D.-A., Lee H., Manson P.N., Elisseeff J. (2005). The effect of incorporating RGD adhesive peptide in polyethylene glycol diacrylate hydrogel on osteogenesis of bone marrow stromal cells. Biomaterials.

[26] Ingavle G.C., Gehrke S.H., Detamore M.S. (2014). The bioactivity of agarose–PEGDA interpenetrating network hydrogels with covalently immobilized RGD peptides and physically entrapped aggrecan. Biomaterials.

[27] DeLong S.A., Moon J.J., West J.L. (2005). Covalently immobilized gradients of bFGF on hydrogel scaffolds for directed cell migration. Biomaterials.

[28] Foudazi R., Zowada R., Manas-Zloczower I., Feke D.L. (2023). Porous hydrogels: Present challenges and future opportunities. Langmuir ACS J. Surf. Colloids.

[29] Nicodemus G.D., Bryant S.J. (2008). The role of hydrogel structure and dynamic loading on chondrocyte gene expression and matrix formation. J. Biomech..

[30] Garg T., Singh O., Arora S., Murthy R. (2012). Scaffold: A novel carrier for cell and drug delivery. Crit. Rev. Ther. Drug Carr. Syst..

[31] Dehghani F., Annabi N. (2011). Engineering porous scaffolds using gas-based techniques. Curr. Opin. Biotechnol..

[32] Keskar V., Marion N.W., Mao J.J., Gemeinhart R.A. (2009). In vitro evaluation of macroporous hydrogels to facilitate stem cell infiltration, growth, and mineralization. Tissue Eng. Part A.

[33] Sannino A., Netti P.A., Madaghiele M., Coccoli V., Luciani A., Maffezzoli A., Nicolais L. (2006). Synthesis and characterization of macroporous poly(ethylene glycol)-based hydrogels for tissue engineering application. J. Biomed. Mater. Res. Part A.

[34] Oliviero O., Ventre M., Netti P.A. (2012). Functional porous hydrogels to study angiogenesis under the effect of controlled release of vascular endothelial growth factor. Acta Biomater..

[35] Beaman H.T., Monroe M.B.B. (2023). Highly porous gas-blown hydrogels for direct cell encapsulation with high cell viability. Tissue Engineering. Part A.

[36] Ju Y.M., Park K., Son J.S., Kim J.-J., Rhie J.-W., Han D.K. (2008). Beneficial effect of hydrophilized porous polymer scaffolds in tissue-engineered cartilage formation. J. Biomed. Mater. Res. Part B Appl. Biomater..

[37] Barbetta A., Gumiero A., Pecci R., Bedini R., Dentini M. (2009). Gas-in-liquid foam templating as a method for the production of highly porous scaffolds. Biomacromolecules.

[38] Poursamar S.A., Hatami J., Lehner A.N., da Silva C.L., Ferreira F.C., Antunes A.P.M. (2015). Gelatin porous scaffolds fabricated using a modified gas foaming technique: Characterisation and cytotoxicity assessment. Mater. Sci. Eng. C Mater. Biol. Appl..

[39] Lim Y.-M., Gwon H.-J., Shin J., Jeun J.P., Nho Y.C. (2008). Preparation of porous poly(ɛ-caprolactone) scaffolds by gas foaming process and in vitro/in vivo degradation behavior using γ-ray irradiation. J. Ind. Eng. Chem..

[40] Nam Y.S., Yoon J.J., Park T.G. (2000). A novel fabrication method of macroporous biodegradable polymer scaffolds using gas foaming salt as a porogen additive. J. Biomed. Mater. Res..

[41] Amani P., Miller R., Javadi A., Firouzi M. (2022). Pickering foams and parameters influencing their characteristics. Adv. Colloid Interface Sci..

[42] Li J., Huang H., Xu T., Li J., Guo T., Lu X., Ren J., Ren X., Weng J. (2022). Effect of the interconnecting window diameter of hydroxyapatite scaffolds on vascularization and osteoinduction. Ceram. Int..

[43] Nguyen Q.T., Hwang Y., Chen A.C., Varghese S., Sah R.L. (2012). Cartilage-like mechanical properties of poly (ethylene glycol)-diacrylate hydrogels. Biomaterials.

[44] Ricka J., Tanaka T. (1984). Swelling of ionic gels: Quantitative performance of the Donnan theory. Macromolecules.

[45] Loh Q.L., Choong C. (2013). Three-dimensional scaffolds for tissue engineering applications: Role of porosity and pore size. Tissue Eng. Part B Rev..

[46] Zhang Q., Lu H., Kawazoe N., Chen G. (2014). Pore size effect of collagen scaffolds on cartilage regeneration. Acta Biomater..

[47] Nava M.M., Draghi L., Giordano C., Pietrabissa R. (2016). The effect of scaffold pore size in cartilage tissue engineering. J. Appl. Biomater. Funct. Mater..

[48] Stenhamre H., Nannmark U., Lindahl A., Gatenholm P., Brittberg M. (2011). Influence of pore size on the redifferentiation potential of human articular chondrocytes in poly(urethane urea) scaffolds. J. Tissue Eng. Regen. Med..

[49] Yamane S., Iwasaki N., Kasahara Y., Harada K., Majima T., Monde K., Nishimura S.-I., Minami A. (2007). Effect of pore size on in vitro cartilage formation using chitosan-based hyaluronic acid hybrid polymer fibers. J. Biomed. Mater. Res. Part A.

[50] Lim S.M., Jang S.H., Oh S.H., Yuk S.H., Im G.I., Lee J.H. (2010). Dual-growth-factor-releasing PCL scaffolds for chondrogenesis of adipose-tissue-derived mesenchymal stem cells. Adv. Eng. Mater..

[51] Ye C., Hu P., Ma M.-X., Xiang Y., Liu R.-G., Shang X.-W. (2009). PHB/PHBHHx scaffolds and human adipose-derived stem cells for cartilage tissue engineering. Biomaterials.

[52] Jang C.H., Koo Y., Kim G. (2020). ASC/chondrocyte-laden alginate hydrogel/PCL hybrid scaffold fabricated using 3D printing for auricle regeneration. Carbohydr. Polym..

[53] Im G.-I., Ko J.-Y., Lee J.H. (2012). Chondrogenesis of adipose stem cells in a porous polymer scaffold: Influence of the pore size. Cell Transplant..

[54] Oh S.H., Kim T.H., Im G.I., Lee J.H. (2010). Investigation of pore size effect on chondrogenic differentiation of adipose stem cells using a pore size gradient scaffold. Biomacromolecules.

[55] Fan H., Hu Y., Zhang C., Li X., Lv R., Qin L., Zhu R. (2006). Cartilage regeneration using mesenchymal stem cells and a PLGA-gelatin/chondroitin/hyaluronate hybrid scaffold. Biomaterials.

[56] Uematsu K., Hattori K., Ishimoto Y., Yamauchi J., Habata T., Takakura Y., Ohgushi H., Fukuchi T., Sato M. (2005). Cartilage regeneration using mesenchymal stem cells and a three-dimensional poly-lactic-glycolic acid (PLGA) scaffold. Biomaterials.

[57] Matsiko A., Gleeson J.P., O’Brien F.J. (2015). Scaffold mean pore size influences mesenchymal stem cell chondrogenic differentiation and matrix deposition. Tissue Eng. Part A.

[58] Xiao Y., He L., Che J. (2012). An effective approach for the fabrication of reinforced composite hydrogel engineered with SWNTs, polypyrrole and PEGDA hydrogel. J. Mater. Chem..

[59] Moura C., Trindade D., Vieira M., Francisco L., Ângelo D.F., Alves N. (2020). Multi-material implants for temporomandibular joint disc repair: Tailored additive manufacturing production. Front. Bioeng. Biotechnol..

[60] Della Sala F., Biondi M., Guarnieri D., Borzacchiello A., Ambrosio L., Mayol L. (2020). Mechanical behavior of bioactive poly(ethylene glycol) diacrylate matrices for biomedical application. J. Mech. Behav. Biomed. Mater..

[61] Christensen R.K., von Halling Laier C., Kiziltay A., Wilson S., Larsen N.B. (2020). 3D printed hydrogel multiassay platforms for robust generation of engineered contractile tissues. Biomacromolecules.

[62] Zhang R., Larsen N.B. (2017). Stereolithographic hydrogel printing of 3D culture chips with biofunctionalized complex 3D perfusion networks. Lab A Chip.

[63] Chan V., Zorlutuna P., Jeong J.H., Kong H., Bashir R. (2010). Three-dimensional photopatterning of hydrogels using stereolithography for long-term cell encapsulation. Lab A Chip.

[64] Dadsetan M., Hefferan T.E., Szatkowski J.P., Mishra P.K., Macura S.I., Lu L., Yaszemski M.J. (2008). Effect of hydrogel porosity on marrow stromal cell phenotypic expression. Biomaterials.

[65] Mohanty S., Larsen L.B., Trifol J., Szabo P., Burri H.V.R., Canali C., Dufva M., Emnéus J., Wolff A. (2015). Fabrication of scalable and structured tissue engineering scaffolds using water dissolvable sacrificial 3D printed moulds. Mater. Sci. Eng. C Mater. Biol. Appl..

[66] Hwang C.M., Sant S., Masaeli M., Kachouie N.N., Zamanian B., Lee S.-H., Khademhosseini A. (2010). Fabrication of three-dimensional porous cell-laden hydrogel for tissue engineering. Biofabrication.

[67] Muniz E.C., Geuskens G. (2001). Compressive elastic modulus of polyacrylamide hydrogels and semi-IPNs with poly(N-isopropylacrylamide). Macromolecules.

[68] Li Z., Liu Z., Ng T.Y., Sharma P. (2020). The effect of water content on the elastic modulus and fracture energy of hydrogel. Extrem. Mech. Lett..

[69] Schinagl R.M., Gurskis D., Chen A.C., Sah R.L. (1997). Depth-dependent confined compression modulus of full-thickness bovine articular cartilage. J. Orthop. Res. Off. Publ. Orthop. Res. Soc..

[70] Park J.S., Chu J.S., Tsou A.D., Diop R., Tang Z., Wang A., Li S. (2011). The effect of matrix stiffness on the differentiation of mesenchymal stem cells in response to TGF-β. Biomaterials.

[71] Steward A.J., Wagner D.R., Kelly D.J. (2013). The pericellular environment regulates cytoskeletal development and the differentiation of mesenchymal stem cells and determines their response to hydrostatic pressure. Eur. Cells Mater..

[72] Otero M., Favero M., Dragomir C., Hachem K.E., Hashimoto K., Plumb D.A., Goldring M.B., Picot J. (2012). Human chondrocyte cultures as models of cartilage-specific gene regulation. Methods in Molecular Biology.

[73] Surmeneva M.A., Kovtun A., Peetsch A., Goroja S.N., Sharonova A.A., Pichugin V.F., Grubova I.Y., Ivanova A.A., Teresov A.D., Koval N.N. (2013). Preparation of a silicate-containing hydroxyapatite-based coating by magnetron sputtering: Structure and osteoblast-like MG63 cells in vitro study. RSC Adv..

[74] Musumeci G., Loreto C., Carnazza M.L., Strehin I., Elisseeff J. (2011). OA cartilage derived chondrocytes encapsulated in poly(ethylene glycol) diacrylate (PEGDA) for the evaluation of cartilage restoration and apoptosis in an in vitro model. Histol. Histopathol..

[75] Musumeci G., Carnazza M.L., Loreto C., Leonardi R., Loreto C. (2012). β-Defensin-4 (HBD-4) is expressed in chondrocytes derived from normal and osteoarthritic cartilage encapsulated in PEGDA scaffold. Acta Histochem..

[76] Johnstone B., Hering T.M., Caplan A.I., Goldberg V.M., Yoo J.U. (1998). In vitro chondrogenesis of bone marrow-derived mesenchymal progenitor cells. Exp. Cell Res..

[77] Markway B.D., Tan G.-K., Brooke G., Hudson J.E., Cooper-White J.J., Doran M.R. (2010). Enhanced chondrogenic differentiation of human bone marrow-derived mesenchymal stem cells in low oxygen environment micropellet cultures. Cell Transplant..

[78] Suzuki S., Muneta T., Tsuji K., Ichinose S., Makino H., Umezawa A., Sekiya I. (2012). Properties and usefulness of aggregates of synovial mesenchymal stem cells as a source for cartilage regeneration. Arthritis Res. Ther..

[79] (2009). Biological Evaluation of Medical Devices—Part 5: Tests for In Vitro Cytotoxicity.

