# Supplementary material for: Fabrication and Characterization of Porous PEGDA Hydrogels for Articular Cartilage Regeneration
Source: Gels. 2024 Jun 26;10(7):422. doi: 10.3390/gels10070422 (PMC11276562; doi:10.3390/gels10070422)
Supplement: Supplementary file 1 [file gels-10-00422-s001.zip › gels-2994295-supplementary.pdf]

Supplementary Information

Fabrication and Characterization of Porous PEGDA Hydrogels for Articular Cartilage Regeneration

Silvia Gonella[1,2,#], Margarida F. Domingues[1,2,#], Filipe Miguel[1,2], Carla S. Moura[3,4,5], Carlos A. V. Rodrigues[1,2], Frederico Castelo Ferreira[1,2] and João C. Silva[1,2,*]

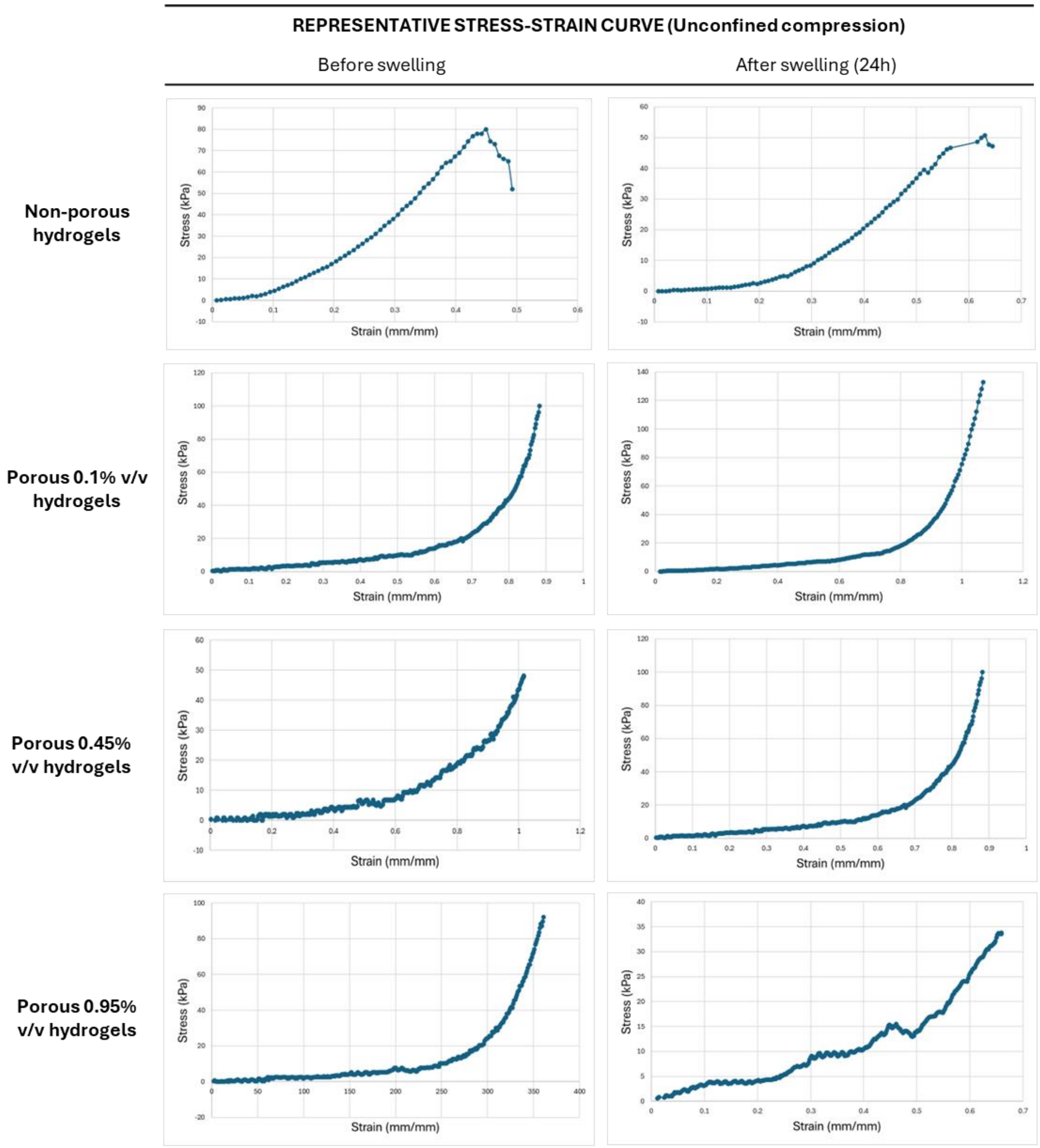

Figure S1 – Representative Stress-Strain curves for an unconfined compression test for 3 conditions of non-porous hydrogels with 0.95% v/v photoinitiator and porous hydrogels with 0.1%, 0.45% or 0.95% v/v photoinitiator concentration, after fabrication and after swelling for 24h.

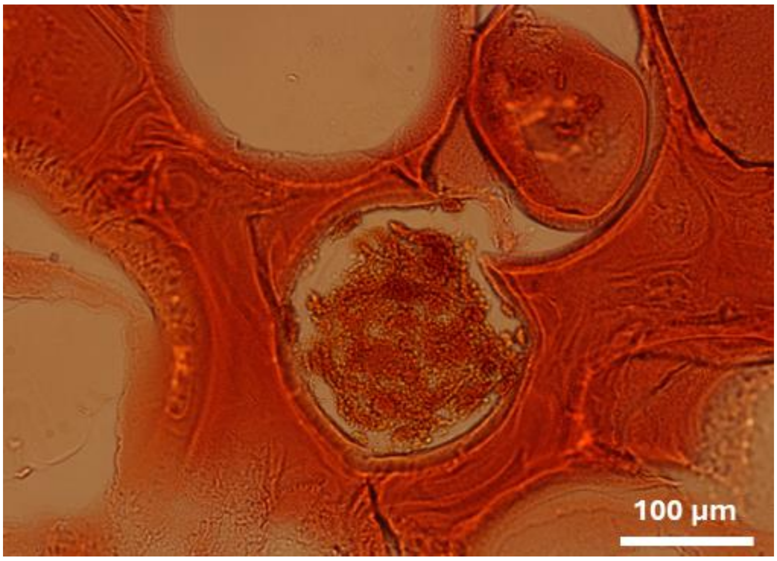

Figure S2 – Safranin-O staining on a 12 µm histological section of the 3D porous PEGDA scaffold seeded with MSC aggregates. Staining of the aggregate was performed after 21 days of culture in chondrogenic medium.

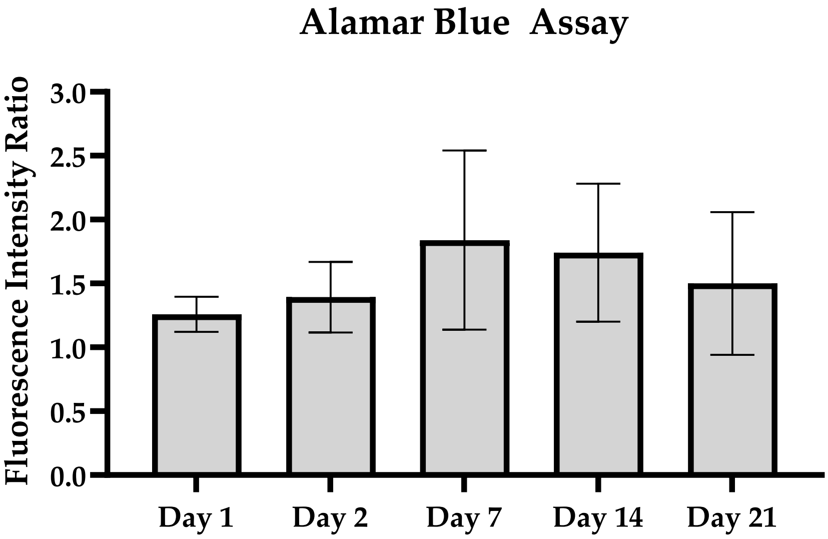

Figure S3 – Alamar Blue assay results for MSC aggregates seeded and cultured on PEGDA porous hydrogel scaffolds at days 1, 2, 7, 14 and 21. The results are presented as the ratio between MSC spheroid-seeded porous hydrogels and acellular porous hydrogels in the respective time points. Error bars represent standard deviation. n=3 independent samples.
